# Evaluating the Antimicrobial Properties of Commercial Hand Sanitizers

**DOI:** 10.1128/mSphere.00062-21

**Published:** 2021-03-03

**Authors:** M. Chojnacki, C. Dobrotka, R. Osborn, W. Johnson, M. Young, B. Meyer, E. Laskey, R. A. F. Wozniak, S. Dewhurst, P. M. Dunman

**Affiliations:** a Department of Microbiology and Immunology, University of Rochester School of Medicine and Dentistry, Rochester, New York, USA; b Department of Ophthalmology, University of Rochester School of Medicine and Dentistry, Rochester, New York, USA; Antimicrobial Development Specialists, LLC

**Keywords:** *Staphylococcus aureus*, *Escherichia coli*, SARS-CoV-2, hand sanitizer

## Abstract

Hand sanitizers have been developed as a convenient means to decontaminate an individual’s hands of bacterial pathogens in situations in which soap and water are not available. Yet to our knowledge, no study has compared the antibacterial efficacy of a large collection of hand sanitizers. Using zone of growth inhibition and kill curve assays, we assessed the performance of 46 commercially available hand sanitizers that were obtained from national chain big-box stores, gasoline stations, pharmacies, and boutiques for antibacterial activity toward prototypical Gram-positive (Staphylococcus aureus) and Gram-negative (Escherichia coli) bacterial pathogens. Results revealed substantial variability in the efficacy of many sanitizers evaluated. Formulations following World Health Organization-recommended ingredients (80% ethanol or 75% isopropyl alcohol) or those including benzalkonium chloride as the active principal ingredient displayed excellent antibacterial activity, whereas others exhibited modest or poor activity in the assays performed. Results also revealed that E. coli was generally more susceptible to most sanitizers in comparison to S. aureus and that there was significant strain-to-strain variability in hand sanitizer antimicrobial efficacy regardless of the organism evaluated. Further, tests of a subset of hand sanitizers toward severe acute respiratory syndrome coronavirus 2 (SARS-CoV-2) revealed no direct correlation between antibacterial and antiviral performance, with all ethyl alcohol formulations performing equally well and displaying improved activity in comparison to benzalkonium chloride-containing sanitizer. Taken together, these results indicate that there is likely to be substantial variability in the antimicrobial performance of commercially available hand sanitizers, particularly toward bacterial pathogens, and a need to evaluate the efficacy of sanitizers under development.

**IMPORTANCE** In response to the coronavirus disease 2019 (COVID-19) pandemic, hand hygiene has taken on a prominent role in efforts to mitigate SARS-CoV-2 transmission and infection, which has led to a radical increase in the number and types of hand sanitizers manufactured to meet public demand. To our knowledge, no studies have evaluated or compared the antimicrobial performance of hand sanitizers that are being produced under COVID-19 emergency authorization. Tests of 46 commercially available hand sanitizers purchased from national chain brick-and-mortar stores revealed considerable variability in their antibacterial performance toward two bacterial pathogens of immediate health care concern, S. aureus and E. coli. Expanded testing of a subset of hand sanitizers revealed no direct correlation between antibacterial performance of individual sanitizers and their activity toward SARS-CoV-2. These results indicate that as the pandemic subsides, there will be a need to validate the antimicrobial efficacy of sanitizers being produced.

## INTRODUCTION

It is well recognized that hand hygiene is essential to reducing microbial burden, transmission, and infection. The density and species of bacteria that colonize the hands of individuals are highly variable and can be influenced by a number of factors including age, sex, ethnicity, and profession (reviewed in reference 1). Health care workers have been of particular interest, as they may provide a reservoir for the circulation and transmission of drug-resistant bacteria within the hospital setting (2). Indeed, studies have revealed that 10.5% to 78.3% of health care workers are colonized with up to 2.4 × 10^7^ per hand of the bacterial pathogen Staphylococcus aureus and may be a source of nosocomial S. aureus infections (reviewed in reference 3). Fortunately, conventional hand washing using water, soap, and friction is an effective means of reducing microbial burden, which when combined with other infection control practices (i.e., glove usage, compliance, and education) has significantly reduced microbial transmission, hospital-acquired infections, reduced gastrointestinal and respiratory illness, and improved overall health (4–6).

In situations in which an individual does not have access to soap and water, the Centers for Disease Control and Prevention (CDC) and World Health Organization (WHO) have recommended the use of alcohol rubs (also known as hand sanitizers) comprised of either 80% ethanol or 75% isopropyl alcohol to reduce microbial burden. Alcohol-based sanitizers have proven to deliver rapid bactericidal activity toward Gram-positive and Gram-negative bacterial pathogens as well as excellent virucidal activity toward both enveloped and nonenveloped viruses of immediate health care concern, including influenza A virus, severe acute respiratory syndrome coronavirus (SARS-CoV), Middle Eastern respiratory syndrome (MERS) virus, Zika virus, Ebola virus, and SARS coronavirus 2 (SARS-CoV-2) (7–10). In response to the SARS-CoV-2 pandemic, there has been increased recognition of the importance of hand hygiene, which has led to an overwhelming increase in hand sanitizer demand. To meet this need, the FDA has released guidance regarding the production of hand sanitizers and temporarily relaxed production restrictions provided that manufacturers follow strict guidelines. This has resulted in an increase in number of products available for public use.

Although data are sparse, previous reports have indicated there can be substantial variability in the antimicrobial performance of individual commercially available hand sanitizers both in *in vitro* testing and when applied to the hands of individuals (11, 12). Yet to our knowledge, there has never been a comparison of the antibacterial properties of a large collection of hand sanitizers; such a study may be timely given the recent onslaught of new sanitizers available on the market. Moreover, to our knowledge, there are not established *in vitro* antimicrobial testing guidelines for sanitizers. Accordingly, herein, we used conventional antibacterial assays to compare the antibacterial performance of 46 commercially available hand sanitizers toward Escherichia coli and Staphylococcus aureus, prototypical Gram-negative and Gram-positive pathogens that are well recognized to contaminate skin surfaces (reviewed in reference 13). We also performed standard antiviral assays to evaluate activity of a subset of sanitizers that were either highly active or had weak activity against S. aureus and/or E. coli toward SARS-CoV-2. Results revealed that there are significant differences in the antibacterial properties of hand sanitizers in the assays used here and also a poor correlation between the antibacterial and antiviral activity of the sanitizers evaluated. From these perspectives, as the current pandemic and corresponding demand for hand rubs subsides, it may be wise to implement formal requirements for antimicrobial efficacy testing of hand sanitizers that have been introduced to the market under emergency COVID-19 authorization to better understand their variability in antibacterial/antiviral efficacy.

## RESULTS

Forty-six hand sanitizers were purchased from national chain big-box stores, gasoline stations, and pharmacies, as well as local boutiques that offer products nationally through e-commerce. Forty-two contained ethyl alcohol (62% to 80%), one contained 75% isopropyl alcohol, and two contained benzalkonium chloride (0.1% to 0.13%) as the active principal antimicrobial ingredient (API). One sanitizer listed 70% alcohol as the API but did not provide additional information regarding the type of alcohol that was included in the formulation. Of the 46 sanitizers, 13 were aqueous or nonviscous in composition, whereas 33 were viscous or gel based in consistency. Each sanitizer was placed in a conical tube and labeled A to Z and AA to AT for testing. A survey of the label ingredients for each sanitizer indicated that samples A and L followed World Health Organization (WHO)-recommended ingredients for ethyl alcohol- and isopropyl alcohol-based sanitizers, respectively, whereas the remainder contained either less alcohol or additional ingredients.

### Staphylococcus aureus plate assay testing.

Each sanitizer was first evaluated for antimicrobial performance toward two S. aureus strains, ATCC 29213 and USA300 (LAC), which are an antibiotic-susceptible laboratory strain and a methicillin-resistant clinical isolate belonging to the USA300 lineage, respectively. The latter was included because the lineage is a predominant cause of U.S. community-associated methicillin-resistant S. aureus (MRSA) infections and, hence, may represent a strain more relevant to health care for testing (14). In parallel, each strain was spread at low density (10^3^ CFU) and high density (10^7^ CFU) onto the surface of an agar plate, a total of 25 μl of sanitizer was applied to the center of the plate (spot plated) and the resulting average zone of growth inhibition (ZOI) was recorded from a total of three independent assays. Additionally, any CFU within the ZOI was enumerated by another means to measure antimicrobial efficacy and/or bacterial sanitizer tolerance.

Low-density spot plate testing revealed differences in the antimicrobial efficacy of the hand sanitizers evaluated. Forty of the 46 sanitizers (87%) produced zones of inhibition ranging in size from ∼6 mm to 40 mm toward both S. aureus strains, indicating that they are effective to different degrees toward the pathogen (Table 1). Six sanitizers that were either formulated following WHO-recommended concentrations of 80% ethanol (three sanitizers) or 75% isopropyl (one sanitizer) or were formulated with benzalkonium chloride (two sanitizers) produced the largest zones of growth inhibition (>20 mm) against both S. aureus strains. Thirty-two sanitizers containing 62% to 78% ethanol generated more modest ZOIs ranging in size from 10 to 19 mm in size toward both strains. Eight sanitizers (62% to 70% ethanol) produced more limited <10-mm zones of inhibition toward at least one of the test strains. Surprisingly, 6 of the 46 (13%) sanitizers tested (62% to 70% ethanol) did not appear to exhibit any antimicrobial effect toward one or both of the S. aureus test strains. More specifically, three sanitizers (AE, AF, and AS) exhibited ∼13.2-mm ZOI toward ATCC 29213 cells but did not exhibit any antimicrobial effect toward strain USA300 (LAC), whereas three sanitizers, N, AA, and R, did not produce any zone of inhibition toward either S. aureus test strain, suggesting that they exhibit lower antistaphylococcal activity in comparison to the other sanitizers evaluated in this study.

**TABLE 1:** Antimicrobial efficacy of 46 hand sanitizers against two S. aureus strains^*a*^

Sample	Active ingredient	Viscosity	S. aureus strain ATCC 29213								S. aureus strain USA300 (LAC) (ZOI)							
			10^3^ CFU plated on MH plate				10^7^ CFU plated on MH plate				10^3^ CFU plated on MH plate				10 ^7^ CFU plated on MH plate			
			ZOI (mm)		No. of CFU within zone		ZOI (mm)		No. of CFU within zone		ZOI (mm)		No. of CFU within zone		ZOI (mm)		No. of CFU within zone	
			Avg	SD	Avg	SD	Avg	SD	Avg	SD	Avg	SD	Avg	SD	Avg	SD	Avg	SD
N	70% ethyl alcohol	Aqueous	**0.0**	**0.0**	**TNTC**	**TNTC**	**0.0**	**0.0**	**TNTC**	**TNTC**	**0.0**	**0.0**	**TNTC**	**TNTC**	**0.0**	**0.0**	**TNTC**	**TNTC**
AA	62% ethyl alcohol	Aqueous	**0.0**	**0.0**	**TNTC**	**TNTC**	**0.0**	**0.0**	**TNTC**	**TNTC**	**14.7**	**5.5**	**TNTC**	**TNTC**	**1.2**	**2.0**	**ND**	**ND**
A	80% ethyl alcohol	Aqueous	40.3	0.6	0.0	0.0	33.0	1.0	0.0	0.0	33.0	1.7	0.0	0.0	17.5	3.1	3.3	3.1
F	63% ethyl alcohol	Aqueous	14.3	1.5	0.3	0.6	**3.7**	**6.4**	**TNTC**	**TNTC**	9.3	1.2	0.3	0.6	**0.0**	**0.0**	**TNTC**	**TNTC**
G	70% ethyl alcohol	Aqueous	15.3	0.6	0.0	0.0	**0.0**	**0.0**	**TNTC**	**TNTC**	15.0	1.0	0.0	0.0	**11.3**	**0.6**	**ND**	**ND**
I	80% ethyl alcohol	Aqueous	32.7	1.2	0.0	0.0	18.3	2.9	12.0	11.1	24.3	1.5	3.3	2.1	**11.7**	**1.2**	**ND**	**ND**
J	0.13% benzalkonium chloride	Aqueous	36.7	3.2	0.0	0.0	36.3	1.5	0.0	0.0	28.0	0.0	0.0	0.0	31.8	3.3	0.0	0.0
L	75% isopropyl alcohol	Aqueous	27.3	0.6	0.0	0.0	15.3	1.5	0.7	0.6	26.0	1.7	3.3	4.9	18.5	1.3	0.0	0.0
R	70% ethyl alcohol	Aqueous	**0.0**	**0.0**	**TNTC**	**TNTC**	**0.0**	**0.0**	**TNTC**	**TNTC**	**0.0**	**0.0**	**TNTC**	**TNTC**	**0.0**	**0.0**	**TNTC**	**TNTC**
AM	75% ethyl alcohol	Aqueous	10.3	0.6	1.0	1.0	**0.0**	**0.0**	**TNTC**	**TNTC**	14.3	1.2	0.0	0.0	**0.0**	**0.0**	**TNTC**	**TNTC**
AQ	80% ethyl alcohol	Aqueous	32.3	1.5	0.0	0.0	18.0	1.0	27.0	38.2	26.3	1.5	0.0	0.0	**12.2**	**2.5**	**ND**	**ND**
AR	62% ethyl alcohol	Aqueous	15.0	0.0	0.0	0.0	**4.7**	**8.1**	**TNTC**	**TNTC**	14.3	0.6	0.0	0.0	**0.0**	**0.0**	**TNTC**	**TNTC**
AT	0.1% benzalkonium chloride	Aqueous	26.0	2.6	0.0	0.0	22.0	1.0	0.0	0.0	22.3	2.5	0.0	0.0	21.8	2.1	0.0	0.0

B	70% ethyl alcohol	Viscous	14.0	1.7	2.3	2.5	**0.0**	**0.0**	**TNTC**	**TNTC**	9.7	0.6	20.0	23.1	**0.0**	**0.0**	**TNTC**	**TNTC**
C	75% ethyl alcohol	Viscous	16.7	3.1	5.0	4.4	6.0	1.7	0.7	0.6	13.7	3.8	0.0	0.0	3.8	0.3	9.7	9.9
D	65% ethyl alcohol	Viscous	15.7	4.7	0.7	0.6	9.3	1.5	0.0	0.0	10.7	1.5	6.3	4.2	10.2	0.6	4.0	2.6
E	75% ethyl alcohol	Viscous	15.7	3.1	0.7	0.6	13.7	1.5	2.3	2.5	11.3	1.2	2.7	3.1	9.7	0.3	1.0	1.7
H	70% alcohol	Viscous	12.0	1.0	0.0	0.0	7.7	1.2	1.0	1.0	9.7	1.5	21.7	21.2	10.3	4.6	8.7	4.9
K	70% ethyl alcohol	Viscous	14.3	1.5	0.7	1.2	6.0	3.0	0.0	0.0	10.3	2.9	4.7	5.5	**8.3**	**2.1**	**ND**	**ND**
M	62% ethyl alcohol	Viscous	6.7	0.6	0.0	0.0	5.3	0.6	0.0	0.0	5.3	0.6	0.0	0.0	5.2	0.3	0.0	0.0
O	62% ethyl alcohol	Viscous	15.3	0.6	0.0	0.0	8.0	0.0	1.0	1.7	13.3	1.2	4.7	1.5	7.7	1.3	12.3	1.5
P	62% ethyl alcohol	Viscous	14.3	4.7	0.0	0.0	**1.7**	**1.5**	**ND**	**ND**	6.0	1.7	10.3	8.4	5.7	1.6	6.7	6.4
Q	70% ethyl alcohol	Viscous	13.0	1.0	0.0	0.0	10.7	0.6	0.0	0.0	13.0	0.0	0.3	0.6	10.2	0.3	0.7	1.2
S	70% ethyl alcohol	Viscous	19.7	0.6	0.0	0.0	17.7	0.6	0.3	0.6	14.3	0.6	0.0	0.0	14.2	2.3	0.0	0.0
T	62% ethyl alcohol	Viscous	12.3	1.5	31.3	28.7	11.0	0.0	0.0	0.0	10.7	0.6	37.0	8.5	**8.0**	**0.0**	**TNTC**	**TNTC**
U	70% ethyl alcohol	Viscous	16.7	0.6	0.0	0.0	15.3	0.6	15.3	21.4	17.7	2.0	0.0	0.0	11.4	2.1	14.3	11.5
V	70% ethyl alcohol	Viscous	17.0	1.0	1.0	1.7	3.7	0.6	0.3	0.6	9.7	0.6	0.0	0.0	8.7	0.6	0.0	0.0
W	65% ethyl alcohol	Viscous	13.7	1.5	0.0	0.0	11.0	0.0	0.3	0.6	11.7	0.6	0.0	0.0	4.8	1.0	1.7	0.6
X	70% ethyl alcohol	Viscous	17.0	1.0	0.0	0.0	9.7	0.6	1.0	1.7	13.0	1.0	2.3	2.1	10.2	2.4	0.7	0.6
Y	62% ethyl alcohol	Viscous	11.3	6.0	0.3	0.6	7.7	1.5	1.0	1.0	15.2	1.4	0.0	0.0	**2.2**	**3.8**	**TNTC**	**TNTC**
Z	68% ethyl alcohol	Viscous	13.0	2.0	0.0	0.0	8.0	1.0	1.0	1.0	5.7	0.6	1.0	1.7	8.8	0.3	0.7	1.2
AB	62% ethyl alcohol	Viscous	16.7	2.1	0.0	0.0	12.7	0.6	1.3	2.3	8.0	6.9	3.0	4.2	9.8	0.3	4.7	1.5
AC	68% ethyl alcohol	Viscous	19.0	2.6	0.0	0.0	15.0	1.0	11.0	7.2	18.0	1.7	0.0	0.0	16.7	1.2	10.3	3.5
AD	65% ethyl alcohol	Viscous	12.3	1.2	1.7	1.5	6.3	0.6	0.0	0.0	10.0	0.0	6.7	4.6	7.2	6.2	3.5	3.5
AE	65% ethyl alcohol	Viscous	14.3	1.2	0.3	0.6	**0.0**	**0.0**	**TNTC**	**TNTC**	**0.0**	**0.0**	**TNTC**	**TNTC**	**0.0**	**0.0**	**TNTC**	**TNTC**
AF	62% ethyl alcohol	Viscous	14.0	0.0	3.3	4.2	**0.0**	**0.0**	**TNTC**	**TNTC**	**10.3**	**0.6**	**TNTC**	**TNTC**	10.2	2.4	17.5	16.3
AG	70% ethyl alcohol	Viscous	17.3	4.5	0.7	1.2	3.3	0.6	1.3	2.3	11.3	3.2	9.7	14.2	9.0	1.7	0.0	0.0
AH	70% ethyl alcohol	Viscous	16.7	4.5	0.0	0.0	11.0	1.0	2.0	2.0	14.0	1.7	0.7	0.6	9.3	0.8	0.0	0.0
AI	70% ethyl alcohol	Viscous	13.0	1.7	0.0	0.0	14.0	2.6	0.0	0.0	14.7	2.3	20.3	24.8	8.3	7.2	0.5	0.7
AJ	78% ethyl alcohol	Viscous	8.3	0.6	0.0	0.0	8.7	0.6	2.7	1.2	10.0	0.0	0.0	0.0	6.3	1.2	13.0	7.2
AK	70% ethyl alcohol	Viscous	14.0	0.0	0.0	0.0	**8.7**	**1.5**	**ND**	**ND**	10.0	0.0	0.0	0.0	9.0	3.6	10.7	6.1
AL	62% ethyl alcohol	Viscous	12.3	2.1	2.3	3.2	7.7	3.1	0.0	0.0	12.7	2.5	0.0	0.0	11.8	1.3	0.0	0.0
AN	62% ethyl alcohol	Viscous	15.3	0.6	0.0	0.0	7.0	2.6	1.7	1.5	14.3	1.2	0.0	0.0	7.2	1.3	2.3	1.5
AO	70% ethyl alcohol	Viscous	13.3	1.2	0.7	0.6	4.7	0.6	0.3	0.6	11.0	0.0	3.3	1.5	6.5	1.3	0.0	0.0
AP	70% ethyl alcohol	Viscous	18.3	1.2	1.0	1.7	**14.7**	**0.6**	**TNTC**	**TNTC**	17.7	5.5	0.0	0.0	**14.8**	**0.6**	**TNTC**	**TNTC**
AS	62% ethyl alcohol	Viscous	11.3	0.6	0.0	0.0	7.7	2.1	2.0	0.0	**0.0**	**0.0**	**TNTC**	**TNTC**	**0.0**	**0.0**	**TNTC**	**TNTC**

Negative control	1.45% glycerin	Aqueous	0.0	0.0	TNTC	TNTC	**0.0**	**0.0**	**TNTC**	**TNTC**	**0.0**	**0.0**	**TNTC**	**TNTC**	**0.0**	**0.0**	**TNTC**	**TNTC**
Positive control	70% ethanol	Aqueous	18.0	0.0	15.0	21.8	**0.0**	**0.0**	**TNTC**	**TNTC**	18.8	0.3	25.0	24.4	**0.0**	**0.0**	**TNTC**	**TNTC**
Positive control	80% ethanol	Aqueous	19.0	1.7	3.3	4.9	**16.3**	**1.2**	**TNTC**	**TNTC**	22.3	2.3	1.3	2.3	16.8	0.3	0.7	1.2

a ZOI, zone of inhibition; TNTC, too numerous to count; ND, too numerous to count in one replicate. Boldface indicates samples that showed no activity.

Spot plate assays using a higher-density inoculum provided further resolution of the comparative antimicrobial performance of each sanitizer toward S. aureus (Table 1). Twenty-seven (59%) sanitizers produced ZOIs ranging from ∼4 mm to ∼32 mm in size toward both strains. Four sanitizers including those formulated following WHO guidelines or with benzylkonium chloride produced the largest zones of inhibition averaging >17 mm toward the two strains, whereas 8 sanitizers produced >10 mm, and 13 sanitizers produced <10-mm ZOIs toward each strain. Conversely, 19 (41%) sanitizers exhibited little to no appreciable antimicrobial activity toward at least one strain. More specifically, each of the six sanitizers that did not exhibit a ZOI at low density also did not exhibit any zone of inhibition in plate assays inoculated with higher density of either S. aureus strain, and 12 hand sanitizers that exhibited appreciable activity toward low inoculum appeared to be less effective toward 10^7^ CFU of one or both of the test strains. Specifically, sanitizer I demonstrated activity toward ATCC 29213 cells but not USA300 (LAC), sanitizers P and AK demonstrated activity toward USA300 (LAC) but no activity toward ATCC 29213, and sanitizers F, G, AM, AR, B, and AE did not exhibit activity toward either strain. Enumeration of colonies that appeared within the zone of inhibition revealed that although sanitizer AP produced a slight 1.3-mm ZOI toward ATCC 29123, the number of colonies that outgrew were too numerous to count. Likewise, sanitizers AQ, K, T, and Y all produced too many CFU within USA300 (LAC) ZOI to count.

Taken together, S. aureus spot plating results revealed that while the majority of hand sanitizers evaluated here displayed appreciable antimicrobial activity toward S. aureus, there was substantial variability in their effectiveness, and several appeared to exhibit little to no activity toward the organism. Results also revealed that there is strain-to-strain variability in the efficacy of individual sanitizers, with some performing well against one test strain, but poorly toward the other and vice versa. Aqueous sanitizers produced a greater ZOI in comparison to viscous agents, even though the latter appeared to readily dissipate during the course of the spot plating assays (results not shown). Indeed, low-density inoculum testing found that the average ZOIs for aqueous sanitizers with a distinguishable zone of inhibition were 25 mm and 21.3 mm toward ATCC 29213 and USA300 (LAC), respectively, whereas viscous sanitizers produced average ZOIs of 14.4 and 11.8 mm toward the two strains. Similarly, at higher cell density, the average ZOIs for aqueous sanitizers were 23.8 mm and 22.4 mm toward strains ATCC 29213 and USA300 (LAC), whereas viscous sanitizers produced average zones of inhibition of 9.2 mm and 8.9 mm, respectively.

### S. aureus kill curve testing.

**FIG 1:**
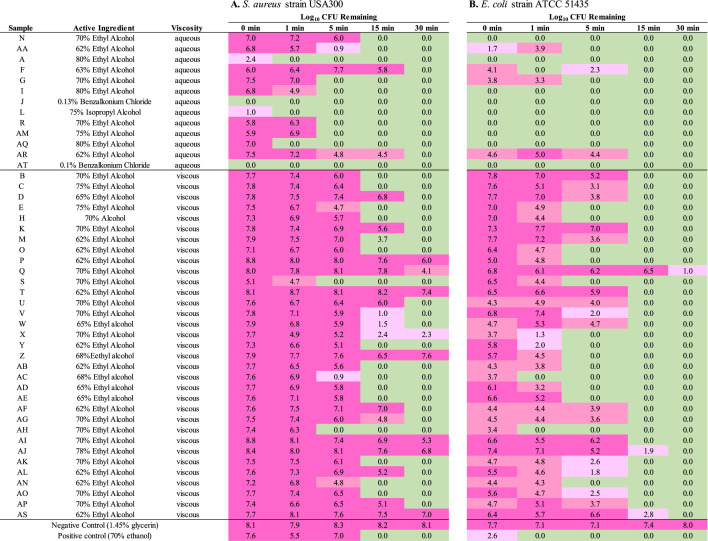
Kill curve data of hand sanitizers toward S. aureus and E. coli. Shown are the log_10_ CFU remaining following 0-, 1-, 5-, 15-, or 30-min treatment of 10^7^ S. aureus strain USA300 (LAC) (A) or E. coli strain ATCC 51435 (B), with the indicated hand sanitizer. Results are averages of three independent assays. Values are shown in color as follows: light green indicates 0 cells, light pink indicates > 0 but < 3 log_10_ cells, pink indicates ≥3 log_10_ but <5 log_10_ cells, and fuchsia indicates ≥5 log_10_ cells remaining.

Kill curve assays were performed as another means to compare the antimicrobial performance of each sanitizer toward S. aureus strain USA300 (LAC). To do so, each sanitizer was mixed directly with an equal volume of 10^7^ CFU of cells, aliquots were collected at various time points, and the average number of CFU remaining was enumerated by plating. Similar to spot plating assays, kill curve results (Fig. 1A) indicated that while most of the sanitizers demonstrated antibacterial activity, there were striking differences in rates of activity among the sanitizers evaluated.

For the aqueous samples, sanitizers containing the WHO-recommended formulations (sample A, 80% ethanol, and sample L, 75% isopropyl alcohol) or benzalkonium chloride (samples J and AT) resulted in either near or total S. aureus eradication within 1 min of cellular challenge, whereas the remainder displayed total bacterial killing at or after 5-min treatment. Sanitizers F and AR, which contained two of the lowest concentrations of ethanol, appeared to demonstrate the most limited antibacterial activity, resulting in 5.8 and 4.5 log_10_ bacteria remaining after 15-min treatment, respectively. Arbitrarily using 1 min as an activity cutoff yielded results that correlated directly with ZOI results. More specifically, aqueous sanitizers that resulted in >6-log_10_-unit decrease in CFU within 1 min of treatment produced the largest zones of inhibition and little to no bacterial outgrowth in high-density spot plating assays (Table 1); conversely, those sanitizers that required greater than 1-min treatment to result in total bacterial kill either failed to demonstrate a ZOI or resulted in significant bacterial outgrowth in high-density S. aureus USA300 (LAC) spot plating.

In comparison to aqueous sanitizers, the gel-based/viscous sanitizers exhibited slower killing kinetics presumably due to limited dissipation of their active principal ingredient, with none demonstrating total bacterial eradication within 1 min of treatment. Survey of the killing kinetics of each of the sanitizers indicated there were clear differences in the rate of bactericidal activity for the viscous sanitizers. Three sanitizers, S, AC, and AH, exhibited the most rapid S. aureus killing properties resulting in near or total USA300 (LAC) killing within 5-min treatment, whereas 45% and 30% of the 33 gel-based sanitizers required 15-min and 30-min treatment to eliminate bacterial cells, respectively. Eight (24%) viscous sanitizers failed to eradicate the bacterial sample during the course of the experiment. Thus, as in the case for the aqueous samples, viscous sanitizers appeared to display different rates of activity, with some acting more quickly than others.

### E. coli plate assay testing.

All sanitizers were also evaluated for antimicrobial activity toward two E. coli strains, the antibiotic-susceptible laboratory strain ATCC 25922 and the drug-resistant clinical isolate ATCC 51435, using spot plating at both low- and high-density inocula. In comparison to S. aureus (above), results revealed that E. coli was generally more susceptible to most of the sanitizers tested, yet there was distinct variability in the antimicrobial performance of the sanitizers (Table 2). More directly, all sanitizers tested produced robust average ZOIs with <5 colonies outgrowth in spot plating assays using low-density inoculum of either E. coli strain. Aqueous sanitizers produced average zones of inhibition of 20.5 mm and 18.6 mm, whereas viscous sanitizers displayed average zones of 15.4 and 13.9 mm toward strains ATCC 25922 and ATCC 51435, respectively. While the majority of the sanitizers evaluated also appeared to display appreciable antimicrobial activity toward high-density inoculum of both E. coli strains, seven sanitizers appeared to be less active. More specifically, sanitizers AQ and B did not appear to be effective toward strain ATCC 25922 but were active toward ATCC 51435, whereas sanitizers AM and AF displayed the opposite phenotype; both were active toward ATCC 25922 but not effective against ATCC 51435. Three sanitizers, AA, R, and P, either failed to display any measurable ZOI or resulted in the outgrowth of too many colonies than could be accurately counted within the zone of inhibition toward both strains. Thus, while all sanitizers appeared to be effective in low-density spot plating assays, there were distinct differences in their efficacy that could be observed in plating assays using higher-density inocula.

**TABLE 2:** Antimicrobial efficacy of 46 hand sanitizers against two E. coli strains^*a*^

Sample	Active ingredient	Viscosity	E. coli strain ATCC 25922								E. coli strain ATCC 51435							
			10^3^ CFU plated on MH plate				10^7^ CFU plated on MH plate				10^3^ CFU plated on MH plate				10 ^7^ CFU plated on MH plate			
			ZOI (mm)		No. of CFU within zone		ZOI (mm)		No. of CFU within zone		ZOI (mm)		No. of CFU within zone		ZOI (mm)		No. of CFU within zone	
			Avg	SD	Avg	SD	Avg	SD	Avg	SD	Avg	SD	Avg	SD	Avg	SD	Avg	SD
N	70% ethyl alcohol	Aqueous	16.2	3.4	0.0	0.0	14.3	1.2	0.0	0.0	16.7	0.6	0.0	0.0	12.7	0.6	0.7	1.2
AA	62% ethyl alcohol	Aqueous	25.0	0.0	0.0	0.0	**17.7**	**1.5**	**ND**	**ND**	17.7	5.9	0.0	0.0	**1.8**	**3.2**	**TNTC**	**TNTC**
A	80% ethyl alcohol	Aqueous	22.0	2.6	0.0	0.0	21.6	2.4	0.0	0.0	20.3	0.6	0.0	0.0	20.3	0.6	0.0	0.0
F	63% ethyl alcohol	Aqueous	16.0	2.2	0.0	0.0	10.7	0.6	0.3	0.6	15.7	2.3	0.0	0.0	13.0	2.6	1.7	0.6
G	70% ethyl alcohol	Aqueous	18.0	2.0	0.0	0.0	11.8	2.8	0.3	0.6	15.0	1.0	0.0	0.0	15.7	0.6	0.0	0.0
I	80% ethyl alcohol	Aqueous	17.3	4.6	0.0	0.0	15.7	2.5	3.0	2.6	21.3	1.5	0.0	0.0	17.0	2.0	2.3	1.2
J	0.13% benzalkonium chloride	Aqueous	35.8	1.4	5.0	4.4	27.0	2.6	0.0	0.0	19.3	0.6	0.0	0.0	19.3	2.1	0.0	0.0
L	75% isopropyl alcohol	Aqueous	26.3	1.2	0.0	0.0	21.7	0.6	0.0	0.0	26.7	1.2	0.0	0.0	25.3	0.6	2.0	2.6
R	70% ethyl alcohol	Aqueous	19.0	0.0	0.7	1.2	**0.0**	**0.0**	**TNTC**	**TNTC**	19.0	1.0	1.3	2.3	**0.0**	**0.0**	**TNTC**	**TNTC**
AM	75% ethyl alcohol	Aqueous	19.7	1.5	0.0	0.0	8.7	2.5	0.0	0.0	14.0	6.1	0.0	0.0	**0.0**	**0.0**	**TNTC**	**TNTC**
AQ	80% ethyl alcohol	Aqueous	22.0	0.0	0.7	1.2	**10.0**	**0.0**	**TNTC**	**TNTC**	20.3	3.1	0.0	0.0	21.3	1.5	0.3	0.6
AR	62% ethyl alcohol	Aqueous	12.2	2.8	0.7	1.2	12.7	0.6	2.0	2.0	14.3	1.2	0.0	0.0	11.3	2.9	1.7	2.9
AT	0.1% benzalkonium chloride	Aqueous	17.0	2.6	0.0	0.0	20.0	5.7	0.0	0.0	22.0	2.0	0.3	0.6	16.3	0.6	0.0	0.0

B	70% ethyl alcohol	Viscous	15.7	1.2	0.0	0.0	**6.7**	**5.8**	**TNTC**	**TNTC**	10.3	2.1	0.0	0.0	7.0	3.5	0.7	1.2
C	75% ethyl alcohol	Viscous	19.0	3.6	0.0	0.0	11.7	6.8	3.3	2.1	9.3	3.8	0.0	0.0	8.7	3.5	0.0	0.0
D	65% ethyl alcohol	Viscous	14.5	3.3	0.0	0.0	10.3	0.6	0.0	0.0	8.3	2.3	0.0	0.0	6.3	2.1	0.0	0.0
E	75% ethyl alcohol	Viscous	15.0	0.0	0.0	0.0	15.3	2.3	0.0	0.0	11.0	3.0	0.0	0.0	15.0	0.0	0.0	0.0
H	70% alcohol	Viscous	14.0	4.4	0.3	0.6	10.2	2.8	0.0	0.0	10.0	4.4	0.0	0.0	11.3	0.6	0.0	0.0
K	70% ethyl alcohol	Viscous	12.8	2.8	0.0	0.0	8.3	3.1	0.3	0.6	14.0	1.7	0.0	0.0	11.0	1.7	0.0	0.0
M	62% ethyl alcohol	Viscous	5.0	0.0	0.0	0.0	5.3	0.6	0.0	0.0	5.0	0.0	0.0	0.0	5.0	0.0	0.0	0.0
O	62% ethyl alcohol	Viscous	19.0	0.0	0.0	0.0	15.7	2.5	0.3	0.6	12.7	4.0	0.0	0.0	11.3	6.4	0.0	0.0
P	62% ethyl alcohol	Viscous	15.0	0.0	0.0	0.0	**0.0**	**0.0**	**TNTC**	**TNTC**	11.7	6.0	0.0	0.0	**0.0**	**0.0**	**TNTC**	**TNTC**
Q	70% ethyl alcohol	Viscous	14.7	1.2	0.0	0.0	9.3	2.1	3.3	3.1	11.3	0.6	0.0	0.0	9.0	2.6	0.0	0.0
S	70% ethyl alcohol	Viscous	13.0	2.6	0.0	0.0	11.7	1.2	0.0	0.0	22.0	1.7	0.0	0.0	17.7	1.2	1.3	2.3
T	62% ethyl alcohol	Viscous	10.0	0.0	0.0	0.0	11.0	0.0	1.3	0.6	15.3	2.5	0.0	0.0	12.7	1.5	0.0	0.0
U	70% ethyl alcohol	Viscous	20.3	0.6	2.7	0.6	12.2	6.2	3.7	4.0	18.0	1.7	0.0	0.0	18.7	2.1	1.7	2.9
V	70% ethyl alcohol	Viscous	17.3	4.0	0.3	0.6	9.7	0.6	0.0	0.0	20.0	2.6	0.0	0.0	7.0	1.7	0.0	0.0
W	65% ethyl alcohol	Viscous	10.0	2.6	0.3	0.6	10.3	0.6	0.0	0.0	15.7	3.1	0.0	0.0	11.3	1.5	0.0	0.0
X	70% ethyl alcohol	Viscous	10.3	0.6	0.0	0.0	9.7	0.6	1.0	1.0	15.7	0.6	0.0	0.0	10.3	1.2	0.7	1.2
Y	62% ethyl alcohol	Viscous	17.7	6.8	0.7	0.6	15.8	3.3	3.0	2.6	17.7	4.9	0.7	0.6	9.0	2.0	1.0	1.0
Z	68% ethyl alcohol	Viscous	13.7	0.6	1.3	1.2	10.8	1.0	0.0	0.0	11.8	2.6	0.0	0.0	11.8	1.6	0.0	0.0
AB	62% Ethyl Alcohol	Viscous	15.0	1.0	0.0	0.0	13.5	1.8	0.0	0.0	16.3	0.6	0.0	0.0	15.7	1.5	0.0	0.0
AC	68% ethyl alcohol	Viscous	21.3	2.3	0.0	0.0	19.7	0.6	1.3	1.2	19.3	0.6	0.0	0.0	17.3	1.2	0.0	0.0
AD	65% ethyl alcohol	Viscous	17.8	4.3	0.0	0.0	6.8	3.5	0.7	1.2	12.7	5.5	0.7	1.2	11.3	2.5	1.0	1.0
AE	65% ethyl alcohol	Viscous	15.3	4.0	0.3	0.6	13.5	2.3	0.3	0.6	15.0	2.6	0.7	1.2	12.5	3.5	0.0	0.0
AF	62% ethyl alcohol	Viscous	16.0	1.0	1.0	1.0	9.0	1.0	1.0	1.7	19.3	1.5	0.3	0.6	**0.0**	**0.0**	**TNTC**	**TNTC**
AG	70% ethyl alcohol	Viscous	19.0	1.7	0.0	0.0	9.2	2.4	1.3	1.5	11.2	5.6	0.0	0.0	8.3	0.6	1.0	1.0
AH	70% ethyl alcohol	Viscous	17.2	1.0	0.0	0.0	14.3	5.5	0.0	0.0	19.0	5.3	0.0	0.0	18.0	0.0	0.3	0.6
AI	70% ethyl alcohol	Viscous	22.2	2.8	0.0	0.0	8.7	0.6	0.0	0.0	10.3	3.8	0.0	0.0	7.7	1.2	0.0	0.0
AJ	78% ethyl alcohol	Viscous	10.0	0.0	0.0	0.0	5.3	0.6	4.3	5.1	9.7	0.6	0.0	0.0	7.0	4.4	1.7	1.5
AK	70% ethyl alcohol	Viscous	16.0	1.0	1.7	2.9	11.3	3.1	0.0	0.0	15.0	0.0	0.0	0.0	12.3	2.9	0.0	0.0
AL	62% ethyl Alcohol	Viscous	17.0	2.6	0.0	0.0	15.2	3.2	0.3	0.6	13.7	3.5	0.0	0.0	11.3	1.5	0.3	0.6
AN	62% ethyl alcohol	Viscous	15.0	0.0	0.0	0.0	8.7	3.2	1.0	1.0	15.3	0.6	0.0	0.0	9.7	2.5	0.0	0.0
AO	70% ethyl alcohol	Viscous	12.8	2.0	0.3	0.6	8.7	1.0	0.3	0.6	11.0	3.5	0.3	0.6	3.7	1.5	0.0	0.0
AP	70% ethyl alcohol	Viscous	18.3	0.6	0.0	0.0	12.3	2.1	0.0	0.0	22.0	1.7	0.0	0.0	16.3	7.2	0.3	0.6
AS	62% ethyl alcohol	Viscous	19.8	0.3	0.3	0.6	10.8	5.3	0.0	0.0	10.0	2.6	1.3	1.2	6.0	5.3	0.0	0.0

Negative control	1.45% glycerin	Aqueous	**0.0**	**0.0**	**TNTC**	**TNTC**	**0.0**	**0.0**	**TNTC**	**TNTC**	**0**	**0**	**TNTC**	**TNTC**	**0.0**	**0.0**	**TNTC**	**TNTC**
Positive control	70% ethanol	Aqueous	22.0	0.0	0.0	0.0	22.0	1.0	1.3	0.6	18.2	0.3	0	0	16.3	1.2	7.7	11.6
Positive control	80% ethanol	Aqueous	16.7	2.1	0.7	0.6	15.3	0.6	5.7	3.5	19.0	3.5	0	0	16.7	4.0	0.3	0.6

a TNTC, too numerous to count; ND, TNTC in at least one replicate. Boldface indicates samples that showed no activity.

### E. coli kill curve testing.

Each sanitizer was also evaluated for antimicrobial activity in kill curve assays using the E. coli clinical isolate ATCC 51435. Results (Fig. 1B) revealed that in comparison to S. aureus (above), E. coli cells were more rapidly eradicated by all of the sanitizers, which corresponds with their increased activity toward E. coli in comparison to S. aureus in spot plate assays. Yet there were striking differences in the rate of bactericidal activity for the agents evaluated. More directly, E. coli cells were totally eradicated by 11 (24%) of the sanitizers within 1 min of treatment, 16 (35%) by 5-min treatment, and 18 (39%) by 15-min treatment, whereas two sanitizers (AS and AJ) required 30-min treatment to totally eradicate cells and sanitizer Q did not display total bactericidal activity at any time point.

Similar to the results of S. aureus testing, the aqueous sanitizers by and large appeared to exhibit more rapid bactericidal activity than viscous sanitizers toward E. coli. Ten (77%) aqueous sanitizers resulted in complete bactericidal activity toward E. coli ATCC 51435 cells within 1-min treatment, whereas only two (6%) of the viscous sanitizers eradicated all cells within 1 min. Yet, unlike with S. aureus testing in which killing kinetics largely corresponded to plate assay results, there were no direct correlations between the E. coli kill curve and plate testing results for the aqueous sanitizers evaluated. For instance, three sanitizers (AA, AM, and R) which failed to generate robust ZOI in high-density plate assays using E. coli strain ATCC 51435 appeared to display rapid bactericidal activity toward the strain in kill curve assays. This disparity could be attributable to nuances of the antimicrobial testing methods, such that sanitizers AA, AM, and R do indeed exhibit rapid bactericidal activity toward cells in direct contact, but adjacent bacteria may overgrow the would-be ZOI in plate assays. It is also noteworthy that these same sanitizers performed poorly against both S. aureus strains, suggesting that they display limited antibacterial activity in comparison to other sanitizers tested.

### SARS-CoV-2 quantitative suspension testing.

Select sanitizers that displayed high (A and J), moderate (N), and low (Q and AI) bactericidal activity were also evaluated for virucidal activity toward the SARS-CoV-2 isolate human hCoV-19/Hong Kong. To do so, eight parts of sanitizer were mixed with one part of organic load, and one part of SARS-CoV-2 suspension for a 1-min contact time and then serially diluted on a monolayer of Vero E6 cells to determine the remaining median tissue culture infectious dose (log_10_ 50% tissue culture infective dose [TCID_50_]/milliliter) after treatment.

**FIG 2:**
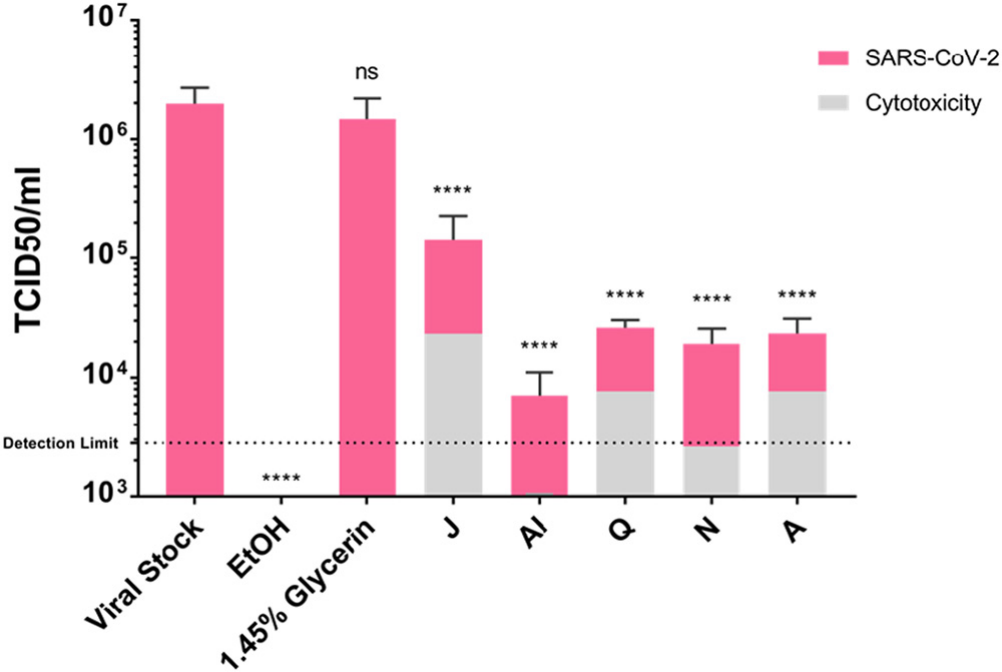
Quantitative SARS-CoV-2 suspension tests. Shown are mean measurements for log_10_ TCID_50_/ml of SARS-CoV-2 isolate hCoV-19/Hong Kong following a 1-min contact time with the indicated hand sanitizer (pink) and sanitizer cytotoxic measures from mock experiments using sanitizers alone (gray). Results are averages of three independent experiments and standard deviations (error bars) are shown; asterisks indicate statistically significant differences in log_10_ TCID_50_/ml compared to the value for the positive control (viral stock without treatment) when data are compared using a one-way ANOVA (****, *P* < 0.0001; ns, not significant). EtOH, ethanol.

As shown in Fig. 2, 1.45% glycerin, a nonactive component of WHO-recommended sanitizer formulations, exhibited no significant virucidal activity toward SARS-CoV-2, whereas 80% ethyl alcohol reduced the viral titer to below detectable limits. Despite differences in their antibacterial performance, viral treatment with each of the four sanitizers (AI, Q, N, and A) containing ethyl alcohol as the API displayed similar reductions (∼2 log_10_ units) in SARS-CoV-2 titers. Further viral treatment with sanitizer J, which contains benzalkonium chloride as the principal active ingredient and exhibited the most potent antibacterial activity, exhibited only a 1-log_10_-unit decrease in SARS-CoV-2 titers under these assay conditions. Importantly, parallel cytotoxicity studies that lacked virus revealed that all of the sanitizers were cytotoxic at lower dilutions than required to measure SARS-CoV-2 cytopathic effects. Taken together, these results suggest that there are no direct correlations between the antibacterial effectiveness of the hand sanitizers tested here toward the bacterial pathogens S. aureus and E. coli and their SARS-CoV-2 antiviral activity. Further, sanitizers formulated with benzalkonium chloride as the principal active ingredient may be less effective in mitigating COVID-19 transmission in comparison to ethyl alcohol-based sanitizers.

## DISCUSSION

Hand sanitizers have been heralded as an effective means to reduce bacterial burden and transmission in situations in which soap and water are unavailable to an individual. Such hand hygiene practices have been accentuated in response to the SARS-CoV-2 pandemic during which the number of commercially available sanitizers has increased radically, and authorities have relaxed stringency for producing sanitizers to meet public demand. To our knowledge, no study has taken a systematic approach toward comparing the antimicrobial performance of a large collection of commercial sanitizers, which was the main goal of our studies.

Using standard spot plating- and kill curve-based assays, we set out to assess the antibacterial properties of sanitizers that are commercially available either by purchase in nationally recognized chain brick-and-mortar stores or by e-commerce. Consequently, it is reasonable to expect that many, if not all, of the sanitizers assessed in this study are currently in use by individuals across the United States. For testing, each sanitizer was evaluated in parallel for activity toward two genetically diverse bacterial species, the Gram-negative pathogen E. coli and the Gram-positive pathogen S. aureus, both of which are common skin contaminants and are a major health care concern. Further, recognizing that antimicrobial agents often display strain specificity, antimicrobial tests were conducted using two strains of each organism—a laboratory-derived antibiotic susceptible strain and a clinical isolate, as the latter may provide more relevant results from a general health care perspective.

Spot plating assays were performed using both low- and high-density plate inocula as a means both to assess the antibacterial effects of each hand sanitizer and to improve resolution in terms of comparing the effectiveness of each sanitizer to one another. Results revealed the following. (i) There was considerable variability in the effectiveness of the individual sanitizers tested. (ii) E. coli generally appeared to be more susceptible to the antimicrobial effects of sanitizers than S. aureus. (iii) There was strain-to-strain variation in the effectiveness of some sanitizers. (iv) High-density inocula provided high resolution in terms of differentiating the effectiveness of sanitizers.

Of the aqueous sanitizers tested, formulations following WHO guidelines (A and L) and those containing benzalkonium chloride as the principal active ingredient (J and AT) appeared to provide the greatest antibacterial activity across all spot plating assays in terms of ZOI size and limited outgrowth within the zone of inhibition, regardless of bacterial species, strain, or inoculum density tested. While most other aqueous formulations exhibited comparable (or even larger) zones of inhibition in low-density spot plate testing, each produced decreased or no zone of inhibition in high-density spot plating assays toward at least one of the four bacterial strains tested. Aqueous sanitizer spot plating results directly correlated with kill curve studies in which each hand sanitizer was evaluated for bactericidal activity toward a high density of S. aureus USA300 cells. Indeed, formulations containing WHO-recommended or benzalkonium chloride formulations nearly or totally eradicated the bacterial inoculum within 1 min of treatment. Conversely, sanitizers that were less active in high-density spot plating required >1-min treatment for bacterial eradication. The strong correlation between spot plate and kill curve results indicates that either assay could be used to effectively compare the antibacterial effects of aqueous sanitizers under development. Further, to ensure comparable activity, it may be worthwhile for manufacturers to ensure that future aqueous sanitizers display comparable antimicrobial efficacy to WHO/benzalkonium chloride formulations to be considered highly effective.

In comparison to aqueous formulations, viscous sanitizers produced both reduced zones of inhibition and slower killing kinetics. Presumably, this is attributable to diminished release of active ingredient(s) as opposed to inactivation of the principal active ingredient, although we cannot rule out either possibility, and virtually all of the viscous sanitizers fully dissipated during the incubation step during both spot plating and kill curve assays. While viscous sanitizers exhibited slow killing kinetics, most were active in spot plating assays. Thus, the latter may represent a better approach for comparing the effectiveness of viscous sanitizers because it does provide a platform to evaluate whether the sanitizer is likely to be effective toward organisms that are in direct contact, with the caveat that spot plating does not allow a measure of the speed of bactericidal activity. A survey of spot plating results for the viscous sanitizers makes it readily apparent that there are significant differences in efficacy among the sanitizers. Indeed, some viscous sanitizers, such as sanitizer S, produced robust zones of inhibition toward all strains of both E. coli and S. aureus with nearly no bacterial outgrowth within the ZOI, indicating that they are highly effective (yet again it is however difficult to estimate how rapidly they work). Conversely, others such as AE produced either no zone of inhibition or too many colonies to count within the ZOI toward one or more of the bacterial strains being evaluated, indicating that they are likely to be less effective antibacterial sanitizers.

Results also revealed that the antibacterial efficacy of some sanitizers are organism and/or strain specific, for reasons that are currently unclear. For instance, sanitizer AE was relatively effective in producing robust ZOIs toward both test E. coli strains but displayed weak activity toward S. aureus strain ATCC 29513 in high-density spot plating and no activity toward S. aureus USA300 (LAC) in either low- or high-density spot plating assays. Thus, while sanitizer AE may be effective in reducing E. coli burden, it is likely to be less effective toward S. aureus in comparison to other sanitizers evaluated. Similarly, strain-to-strain variability was also noted for some sanitizers. For instance, sanitizer AS displayed activity toward S. aureus strain ATCC 29513 in both low- and high-density spot plating assays, but the sanitizer was not active toward S. aureus strain USA300 (LAC) in spot plating or kill curve testing. Likewise, sanitizer AF was effective in both low- and high-density spot plating assays using E. coli strain ATCC 25922 but had no measurable activity toward E. coli strain ATCC 51435 in high-density spot plating. From these perspectives, future sanitizer development campaigns should consider evaluating their products for activity toward multiple bacterial species and strains for antimicrobial efficacy.

We also evaluated the performance of select hand sanitizers that displayed either low, modest, or high S. aureus and/or E. coli antibacterial activities toward SARS-CoV-2 and found no direct correlation between antibacterial and antiviral activity. Rather, results revealed that the ethyl alcohol-based sanitizers appeared equipotent to one another, resulting in ∼2-log_10_-unit decrease in SARS-CoV-2 (Hong Kong) titer. The observed reduction in SARS-CoV-2 (Hong Kong) in our study for sanitizer A was less than the reduction previously reported by Kratzel and colleagues (>3 log_10_ units) for 80% WHO ethyl alcohol-based formulation toward SARS-CoV-2 (München) (7). The difference in efficacy between the two studies could be attributable to SARS-CoV-2 strain variability, unrecognized variability in preparation of the two WHO formulations, and/or differing methods of reporting antiviral effects. With regard to the latter, Kratzel and colleagues smartly took into account the cytotoxic effects of hand sanitizers by calculating a reduction factor (RF), yet we were unable to fully reconcile whether that study presented RF calculations as virucidal activity. In the current study, we report both sanitizer virucidal and cytotoxic measures. Interestingly, results of our study also revealed that sanitizer containing benzalkonium chloride was slightly less effective toward the virus than ethyl alcohol-based formulations.

Taken together, the results of this study suggest that not all hand sanitizers are equally effective bactericidal agents toward E. coli and S. aureus, as judged by the assays performed. As noted, some sanitizers appeared effective toward one or both organisms, whereas the antibacterial effects of other sanitizers seemed to wane. Further, there may be minor, yet appreciable, differences in efficacy of benzalkonium chloride and ethyl alcohol-based formulations toward SARS-CoV-2. Thus, as the COVID-19 pandemic subsides in the United States, it may be wise to implement formal requirements for efficacy data as a requisite for the continued production of hand sanitizers that have been introduced to the market under emergency COVID-19 authorization. Results of these studies indicate that antibacterial testing should probably be conducted using multiple species and strains and could be performed using either high-density spot plating and/or kill curve assays for aqueous sanitizers, whereas kill curve studies are less revealing for viscous sanitizers. Similarly, it may be important to evaluate the effectiveness of sanitizers toward multiple strains of SARS-CoV-2.

## MATERIALS AND METHODS

### Hand sanitizers.

Forty-six commercial hand sanitizers were purchased from local (Rochester, NY) national chain big-box stores, gasoline stations, and pharmacies as well as boutiques that provide products throughout the country via e-commerce. Aliquots of each were placed in 15-ml conical tubes and labeled A to Z and AA to AT to blind researchers. Each sample was tested for antimicrobial performance using two standard antimicrobial assays—spot plating and kill curve, as described below.

### Bacterial strains used in these studies.

Two strains of each organism were selected for testing—an antibiotic-susceptible strain and an antibiotic-resistant clinical isolate based on the premise that the latter may be more biologically relevant. With the exception of S. aureus strain USA300 (LAC), all strains were directly purchased from the American Type Culture Collection (ATCC) for this project. E. coli ATCC 25922 is an antibiotic-susceptible strain that is recommended for antimicrobial testing by the ATCC (ATCC catalog no. 25922), whereas E. coli ATCC 51435 is an antibiotic-resistant clinical isolate (ATCC catalog no. 51435). S. aureus ATCC 29213 is an antibiotic-susceptible strain that is recommended for antimicrobial testing by the ATCC (ATCC catalog no. 29213), whereas the USA300 strain used here has been previously characterized and is representative of strains causing community-acquired MRSA infections within the United States (15).

### Virus and cell line used in these studies.

SARS coronavirus 2 (SARS-CoV-2) isolate human hCoV-19/Hong Kong/VM20001061/2020 (BEI catalog no. NR-52282) was purchased directly from the Biodefense and Emerging Infections Research Resources Repository (BEI Resources; Manassas, VA). SARS-CoV-2 was propagated and the titers of the virus were determined in African green monkey kidney epithelial Vero E6 cells (American Type Culture Collection; Manassas, VA) in Eagle minimum essential medium (Lonza; Basel Switzerland) supplemented with 2% fetal bovine serum (FBS) (Atlanta Biologicals), 2 mM l-glutamine (Lonza), and 1% penicillin (100 U/ml) and streptomycin (100 μg/ml). Virus stock was stored at − 80°C. All work involving SARS-CoV-2 was performed in the CDC/USDA-approved biosafety level 3 (BSL-3) core facility of the University of Rochester School of Medicine and Dentistry in accordance with institutional biosafety requirements.

### Spot plating assays.

Bacteria were grown overnight and subcultured in fresh Mueller-Hinton medium at 37°C with aeration. In duplicate, a total of 10^3^ or 10^7^ CFU was spread onto the surface of a Mueller-Hinton agar plate. The plate was dried for 5 min, and 25 μl of sanitizer was directly applied (spot) onto the center of the plate surface and then incubated overnight at 37°C to allow for bacterial growth. The antimicrobial performance of each sanitizer was measured as the diameter of the sanitizer-associated zone of bacterial growth inhibition in millimeters. Each species and strain were tested in triplicate for plates inoculated with both 10^3^ and 10^7^ CFU. The average zone of inhibition (ZOI) and standard deviation (SD) were recorded. In addition, colonies within the ZOI were counted and recorded to serve as an additional measure of antimicrobial performance and/or potential bacterial tolerance to each sanitizer.

### Kill curve assays.

Bacteria were grown overnight and subcultured in fresh Mueller-Hinton medium at 37°C with aeration. A total of 10^7^ CFU ml^−1^ of the indicated bacterial species and strain was mixed (1:1) with hand sanitizer. Aliquots were removed at 0, 1, 5, 15, and 30 min after treatment, serially diluted in sterile saline, and plated to enumerate the number of bacteria ml^−1^ remaining. E. coli strain ATCC 51435 and S. aureus strain USA300 (LAC) were tested in triplicate, and the average log_10_ bacteria remaining was recorded. Of note, pilot tests of various bacterium-to-sanitizer ratios (0.25:1, 0.5:1, and 1:1 [vol/vol]) were initially performed; 1:1 ratio provided the greatest dynamic range between the negative control (1.45% glycerin) and the positive control (70% ethanol) and hence was selected for testing the entire sanitizer collection.

### Quantitative suspension assay.

All antiviral experiments were performed in accordance with prEN 14476:2011 (16). Briefly, for each sanitizer, eight parts of sanitizer were mixed with one part of organic load (7.5% bovine serum albumin [BSA] fraction V [ThermoFisher Scientific, Waltham, MA]) and one part of SARS-CoV-2 viral stock. After a 1-min contact time, the mixture was diluted 1:10 with infection medium. Viral titers were then determined using the tissue culture infectious dose assay on triplicate wells of an 80% confluent monolayer of Vero E6 cells in a 96-well microtiter plate format using a 1:3 dilution factor following 4 days of incubation at 37°C in a CO_2_ incubator and microscopic examination of cytopathic effects. Infectious dose (log_10_ TCID_50_/ml) was calculated using the Spearman-Kärber method (17). In parallel, sanitizer cytotoxicity was quantified in the absence of virus through observed changes in cell density and morphology and calculated using the Spearman-Kärber method.

### Statistical analysis.

The mean log_10_ TCID_50_/ml and standard deviations were quantified from three biological replicates. Reduction factors (RFs) for each treatment condition were calculated using the difference of the log_10_ SARS-CoV-2 treatment and sanitizer cytotoxicity mean log_10_ TCID_50_/ml. We compared mean log_10_ TCID_50_/ml using a one-way analysis of variance (ANOVA) procedure with Prism version 7.0.2 (GraphPad Software, San Diego, CA).

## References

[1] Rosenthal M, Goldberg D, Aiello A, Larson E, Foxman B. 2011. Skin microbiota: microbial community structure and its potential association with health and disease. Infect Genet Evol 11:839–848. doi:10.1016/j.meegid.2011.03.022. 21463709 PMC3114449

[2] Aiello AE, Cimiotti J, Della-Latta P, Larson EL. 2003. A comparison of the bacteria found on the hands of 'homemakers' and neonatal intensive care unit nurses. J Hosp Infect 54:310–315. doi:10.1016/S0195-6701(03)00146-4. 12919763 PMC2062569

[3] Kampf G, Kramer A. 2004. Epidemiologic background of hand hygiene and evaluation of the most important agents for scrubs and rubs. Clin Microbiol Rev 17:863–893, table of contents. doi:10.1128/CMR.17.4.863-893.2004. 15489352 PMC523567

[4] Aiello AE, Coulborn RM, Perez V, Larson EL. 2008. Effect of hand hygiene on infectious disease risk in the community setting: a meta-analysis. Am J Public Health 98:1372–1381. doi:10.2105/AJPH.2007.124610. 18556606 PMC2446461

[5] Burton M, Cobb E, Donachie P, Judah G, Curtis V, Schmidt W-P. 2011. The effect of handwashing with water or soap on bacterial contamination of hands. Int J Environ Res Public Health 8:97–104. doi:10.3390/ijerph8010097. 21318017 PMC3037063

[6] Sickbert-Bennett EE, DiBiase LM, Willis TMS, Wolak ES, Weber DJ, Rutala WA. 2016. Reduction of healthcare-associated infections by exceeding high compliance with hand hygiene practices. Emerg Infect Dis 22:1628–1630. doi:10.3201/eid2209.151440. 27532259 PMC4994356

[7] Kratzel A, Todt D, V'kovski P, Steiner S, Gultom M, Thao TTN, Ebert N, Holwerda M, Steinmann J, Niemeyer D, Dijkman R, Kampf G, Drosten C, Steinmann E, Thiel V, Pfaender S. 2020. Inactivation of severe acute respiratory syndrome coronavirus 2 by WHO-recommended hand rub formulations and alcohols. Emerg Infect Dis 26:1592–1595. doi:10.3201/eid2607.200915. 32284092 PMC7323537

[8] Larson EL, Cohen B, Baxter KA. 2012. Analysis of alcohol-based hand sanitizer delivery systems: efficacy of foam, gel, and wipes against influenza A (H1N1) virus on hands. Am J Infect Control 40:806–809. doi:10.1016/j.ajic.2011.10.016. 22325728

[9] Rabenau HF, Kampf G, Cinatl J, Doerr HW. 2005. Efficacy of various disinfectants against SARS coronavirus. J Hosp Infect 61:107–111. doi:10.1016/j.jhin.2004.12.023. 15923059 PMC7132504

[10] Siddharta A, Pfaender S, Vielle NJ, Dijkman R, Friesland M, Becker B, Yang J, Engelmann M, Todt D, Windisch MP, Brill FH, Steinmann J, Steinmann J, Becker S, Alves MP, Pietschmann T, Eickmann M, Thiel V, Steinmann E. 2017. Virucidal activity of World Health Organization-recommended formulations against enveloped viruses, including Zika, Ebola, and emerging coronaviruses. J Infect Dis 215:902–906. doi:10.1093/infdis/jix046. 28453839 PMC5407053

[11] Jain V, Karibasappa G, Dodamani A, Prashanth VK, Mali G. 2016. Comparative assessment of antimicrobial efficacy of different hand sanitizers: an in vitro study. Dent Res J 13:424–431. doi:10.4103/1735-3327.192283. PMC5091001 27857768

[12] Reynolds SA, Levy F, Walker ES. 2006. Hand sanitizer alert. Emerg Infect Dis 12:527–529. doi:10.3201/eid1203.050955. 16710985 PMC3291447

[13] Scott E. 2013. Community-based infections and the potential role of common touch surfaces as vectors for the transmission of infectious agents in home and community settings. Am J Infect Control 41:1087–1092. doi:10.1016/j.ajic.2013.05.014. 23973421

[14] McDougal LK, Steward CD, Killgore GE, Chaitram JM, McAllister SK, Tenover FC. 2003. Pulsed-field gel electrophoresis typing of oxacillin-resistant Staphylococcus aureus isolates from the United States: establishing a national database. J Clin Microbiol 41:5113–5120. doi:10.1128/jcm.41.11.5113-5120.2003. 14605147 PMC262524

[15] Miller LG, Perdreau-Remington F, Rieg G, Mehdi S, Perlroth J, Bayer AS, Tang AW, Phung TO, Spellberg B. 2005. Necrotizing fasciitis caused by community-associated methicillin-resistant Staphylococcus aureus in Los Angeles. N Engl J Med 352:1445–1453. doi:10.1056/NEJMoa042683. 15814880

[16] Steinmann J, Becker B, Bischoff B, Magulski T, Steinmann J, Steinmann E. 2013. Virucidal activity of Formulation I of the World Health Organization’s alcohol-based handrubs: impact of changes in key ingredient levels and test parameters. Antimicrob Resist Infect Control 2:34–34. doi:10.1186/2047-2994-2-34. 24330802 PMC4029200

[17] Hierholzer J, Killington R. 1996. Virus isolation and quantitation, p 25–46. *In* Mahy BWJ, Kangro HO (ed), Virology methods manual. Academic Press, San Diego, CA.

